# Estimated annual costs of Chikungunya fever in the municipality of Rio de Janeiro, Brazil

**DOI:** 10.1590/1980-549720240026

**Published:** 2024-06-14

**Authors:** Thauanne de Souza Gonçalves, Cleber Nascimento do Carmo, Daniel Savignon Marinho

**Affiliations:** I Universidade do Estado do Rio de Janeiro, Institute of Social Medicine – Rio de Janeiro (RJ), Brazil.; II Fundação Oswaldo Cruz, National School of Public Health – Rio de Janeiro (RJ), Brazil.

**Keywords:** Chikungunya fever, Chikungunya virus, Costs and cost analysis, Cost of illness

## Abstract

**Objective:**

To estimate the cost of illness of Chikungunya in the municipality of Rio de Janeiro, Brazil, in 2019.

**Methods:**

The study is a partial economic evaluation carried out with secondary data with free and unrestricted access. Direct outpatient and indirect costs of the acute, post-acute, and chronic phases of Chikungunya fever were estimated, in addition to hospital costs. The estimate of direct costs was performed using the notified cases and the standard treatment flowchart in the state of Rio de Janeiro. The indirect ones consist of loss of productivity and disability, using the burden of disease indicator (Disability-adjusted life year – DALY).

**Results:**

The total number of reported cases was 38,830. Total costs were calculated at BRL 279,807,318, with 97% related to indirect costs.

**Conclusion:**

The chronic phase and indirect costs were the most expensive. The inability and permanence of Chikungunya differentiate the disease and increase the costs of its treatment.

## INTRODUCTION

Chikungunya fever (CHIKF) is an arbovirus transmitted by the bite of female mosquitoes of the genus *Aedes*, described by Johann Wilhelm Meigen in 1818. The disease emerged in Brazil in 2014 and added Zika, dengue, and yellow fever to the list of diseases caused by arboviruses of epidemiological importance. Currently, there are autochthonous transmission records in all Brazilian states^1^.

In 2019, the Notifiable Diseases Information System (*Sistema de Informação de Agravos de Notificação* – Sinan) registered 178,500 probable cases in the country. The Southeast region had the highest incidence, with 113,607 reported cases (64%), 140 per 100 thousand inhabitants, while the national rate was 87.9 cases per 100 thousand inhabitants. Although CHIKF notifications in the following years in Brazil remained above 100 thousand cases, reaching 270 thousand in 2022, 2019 registered the most cases in the Southeast region, with three to five times more notifications than in the following years.

Most cases of CHIKF virus infection have clinical manifestations and may develop in up to three phases: acute, lasting between five and 14 days; post-acute, lasting up to three months; and chronic, with symptoms lasting longer than three months. This characteristic sets CHIKF apart from other arboviruses^2^.

In the years 2016 and 2017, authors of a study estimated the burden of CHIKF in Brazil. In 2016, considering 236,415 cases and 301 deaths, the disability-adjusted life years (DALY) were 77,422.61, equivalent to 0.3757 per thousand inhabitants, with an absolute predominance of those lost years being caused by cases in the chronic phase. Likewise, in 2017, with 181,882 cases and 250 deaths, there were 59,307.59 DALYs or 0.2856 per thousand inhabitants^3^.

The case fatality rate caused by CHIKF is low when compared to other diseases caused by arboviruses. Considering the evolution of the cases available in Sinan, between 2017 and 2022, 603 cases out of the 1,046,035 that were reported during the period progressed to death by CHIKF, which represents a case fatality rate of 0.058%. However, researchers suggested that deaths from CHIKF are underreported and estimated that the case fatality rate may be up to seven times higher than that found in Sinan^4^.

Authors of previous studies carried out during or after CHIKF epidemics identified the relevance of the percentage of cases that remain symptomatic many months after the acute phase, from the first outbreaks that occurred on the African continent between the 1950s and the 1970s to the great epidemic on Réunion in the years 2005 and 2006^5-8^. The latter is noteworthy because of the large number of scientific publications produced after the disease infecting about 40% of the population. Percentages of persistence of arthralgia above 50% were identified between 3 and 18 months after the onset of symptoms in different studies^9-11^.

A method usually employed to measure the impact that diseases cause on healthcare systems and society in general is to estimate their economic costs. Cost-of-illness studies constitute a partial economic assessment that describes the economic impact of a given condition in a defined region^12,13^.

Authors of studies that evaluated the economic impact of CHIKF during periods of outbreaks or epidemics on Réunion, India, Colombia, and the American Virgin Islands calculated amounts ranging from 12 to 76 million dollars in direct and indirect costs related to these outbreaks. The indirect costs are more expensive, related to loss of productivity or disability caused mainly by arthralgia, which may be prolonged for months after the acute phase^14-17^.

The municipality of Rio de Janeiro, Brazil, had its first records of local CHIKF transmission in 2015. During the same period, the city was facing Zika virus outbreaks, which made arbovirus cocirculation an even more relevant issue for the region^18^. In 2016, the estimated costs for CHIKF and Zika in the state of Rio de Janeiro reached BRL 21 million^19^.

The year 2019, in turn, was marked by an outbreak of CHIKF in the municipality of Rio de Janeiro, which represented an increase of more than 300% in the number of reported cases compared to the previous year. The first outbreak in the municipality occurred in 2016, with 14,962 cases reported in Sinan. The year 2017 registered 1,698 cases, and in the following year there was a new increase, reaching 11,140 notifications. In 2019, in turn, there was the highest number of notifications since the emergency of the disease in the area, 38,830. In the following years, between 2020 and 2023, notifications remained between 210 and 931 cases. Thus, the objective of this study was to estimate the cost of illness of CHIKF in the municipality of Rio de Janeiro in the year 2019.

## METHODS

The present study can be classified as a partial cost-of-illness economic assessment based on the CHIKF cases reported during 2019 in the municipality of Rio de Janeiro, Brazil. All reported cases were included in this study, as 99.8% recorded laboratory or clinical-epidemiological confirmation. The municipal population is the second largest in the country, estimated by the 2022 Census at over 6.2 million inhabitants.

Data related to the notifications were obtained from Sinan. The population data were obtained from the 2022 Census of the Brazilian Institute of Geography and Statistics (IBGE). Considering the lack of data on the post-acute and chronic phases, the numbers of cases were estimated using the percentages presented in the scientific literature. All data used are public and freely accessible; hence, they do not require evaluation by a research ethics committee.

To estimate the total costs, the specificities of each phase of the disease were considered, which were individually calculated. The final sum is the result of adding the direct and indirect outpatient costs of the three phases, mortality costs, and hospital costs. Direct costs were estimated based on the determination of the standard outpatient treatment protocol defined by the Rio de Janeiro State Department of Health (*Secretaria de Estado de Saúde do Rio de Janeiro* – SES/RJ)^20^, also available in the clinical management instructions of the Brazilian Ministry of Health^2^. Indirect costs represent lost of productivity and were estimated using the human capital method. This method considers as costs the hours of productivity lost due to illness, considering the possible wage losses^21^. The costs of mortality compose the indirect costs. All data used are described in Table 1 ^22-29^.

**Table 1:** Description of the used data and their sources.

Estimated data	Amount	Amount used	Source
Medical appointment	Average monthly wage: BRL 14,830.18 or BRL 92.69 per hour	Medical appointment (15 min): BRL 23.17	Public notices^22-24^
Total cases of acute phase/notifications	N0=38,830	N0=38,830	Sinan-Rio^25^
Total cases of post-acute phase	53.7% of N0	20,852	Rodriguez-Morales et al.^26^
Total cases of chronic phase	52% of N0	20,192	Edington et al.^27^
Dipyrone 500 mg	Mean: BRL 0.0774	BRL 0.0774	BPS, 2019^28^
Acetaminophen 500 mg	Mean: BRL 0.0404	BRL 0.0404	BPS, 2019^28^
Tramadol 50 mg	Mean: BRL 0.1514	BRL 0.1514	BPS, 2019^28^
Codeine 30 mg	Mean: BRL 0.7482	BRL 0.7482	BPS, 2019^28^
Oxycodone 10 mg	Mean: BRL 6.3804	BRL 6.3804	BPS, 2019^28^
Prednisone 20 mg	Mean: BRL 0.1454	BRL 0.1454	BPS, 2019^28^
Ibuprofen 600 mg	Mean: BRL 0.1917	BRL 0.1917	BPS, 2019^28^
Amitriptyline 25 mg	Mean: BRL 0.0379	BRL 0.0379	BPS, 2019^28^
Gabapentin 300 mg	Mean: BRL 0.3273	BRL 0.3273	BPS, 2019^28^
Hydroxychloroquine 400 mg	Mean: BRL 1.3599	BRL 1.3599	BPS, 2019^28^
Methotrexate 2.5 mg	Mean: BRL 0.7629	BRL 0.7629	BPS, 2019^28^
Folic acid 5 mg	Mean: BRL 0.0362	BRL 0.0362	BPS, 2019^28^
GDP per capita (2020)	BRL 49,094.40 (annual)	BRL 134.51	IBGE^29^
Life expectancy at birth (Brazil, 2019)	76.6 years	76.6 years minus age of death	IBGE^29^

### Direct costs

**Figure 1:**
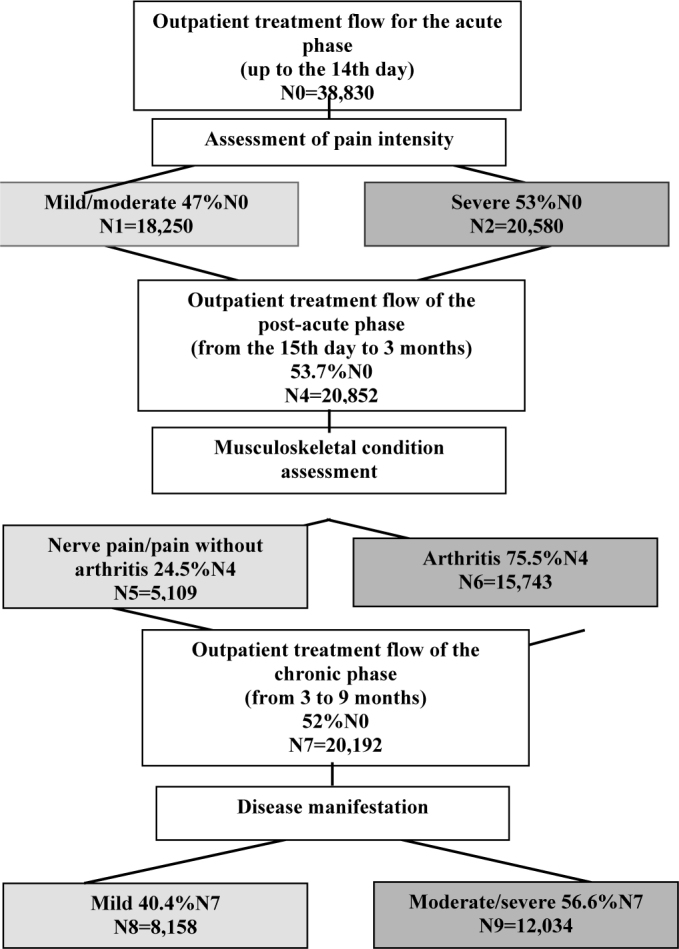
Expected cases of Chikungunya in 2019 in the municipality of Rio de Janeiro, by categories of pain intensity, musculoskeletal condition, and disease manifestation, respectively related to the treatment of acute, post-acute, and chronic phases

The treatment flow, with the estimated number of cases for the three phases of the disease, is demonstrated in Figure 1. The duration of the acute phase is up to 14 days. All reported cases were considered symptomatic, as the criteria for notification involves acute joint pain with a sudden onset; therefore, the authors considered that the number of cases that underwent outpatient treatment in the acute phase is equivalent to that of notifications^2^. In this first phase, it is estimated that each patient had two medical appointments and that their prescription was made according to the intensity of their pain. The percentage of cases with minor/moderate or severe pain was considered to be 47 and 53%, respectively, as described in the study by Delgado-Enciso et al.^30^

Hence, the persistence of symptoms starting on the 15th day prompts individuals to continue outpatient treatment. It was considered that 53.7% of the incident cases remained symptomatic for three months, as described by Rodriguez-Morales et al.^26^ The expectation of prescribing outpatient treatment is divided between the types of musculoskeletal manifestations, whether with or without arthritis, and the expected number of cases in each category is 75.5 and 24.5%, respectively, as indicated by Marques et al.^31^ During the period, three medical appointments are expected per patient^20^.

The persistence of symptoms beyond three months places the patient into the treatment flow for the chronic phase. In this case, the used protocol recommends maintaining this outpatient treatment for up to six months; then, if symptoms persist, the indication is for referral for rheumatological follow-up. Therefore, in this article, the six-month duration was considered for the treatment of the chronic phase^20^. It is estimated that 52% of the reported cases progress to the chronic phase, as described in the meta-analysis carried out by Edington et al.^27^ Three medical appointments are expected, and the prescription of the medication is defined according to the intensity of the manifestation of the disease. The expected percentage is 40.4% of low activity and 59.6% of moderate/intense activity, as described by Abella et al.^32^

To define the medication prices, the prices for the year 2019, which were accessed during the year 2020, were considered. All medication purchase prices are derived from the Health Price Bank of the Ministry of Health (*Banco de Preços em Saúde* – BPS) portal, in which the price obtained by the procurement was considered, when prices are available for the state of Rio de Janeiro. To estimate the cost of medications, the average of the prices available in the BPS was calculated, after excluding values that were greater or lower than 1.5 times the interquartile range of the average, to avoid price distortion by non-standard procurements.

The price of the medical appointment was estimated based on the average amount of the wage available in three professional hiring notices issued in the years 2019 and 2020^22-24^. The monthly wage was divided by the weekly 40 hours, and based on the amount of the working hour, it was estimated how much each appointment with an average duration of 15 minutes would cost.

### Indirect costs

The estimate of indirect costs considered the human capital method, which considers the patient’s perspective and their non-working hours as financial losses^21^.

For the indirect costs of the acute phase, simple productivity loss was estimated, considering the recommendation of a seven-day medical certificate^2^. Therefore, the financial loss was calculated according to the gross domestic product per capita per day (GDPpcday) of the municipality of Rio de Janeiro, according to the following equation: Eq. 1

GDPpcday×seven days×N. (1)

For the indirect costs related to the post-acute and chronic phases, the burden of disease indicator, DALY, was used, which represents the lost disability-adjusted life years and is equivalent to the sum of the years of life lost (YLL) and the years lived with disability (YLD)^33^. Based on this indicator, the indirect costs of persisting symptoms were estimated, considering that DALYs are equivalent to total years of loss of productivity. Other researchers have also proposed this type of methodology that combines disease burden estimates with indirect costs^19,34^. The following equation represents the calculation for the lost of productivity costs of the post-acute and chronic phases: Eq. 2

Municipal per capita GDP×DALY. (2)

### Hospital costs

Hospital costs are derived from hospitalization authorizations (HAAs) provided by the Hospital Information System of the Brazilian Unified Health System (*Sistema de Informações Hospitalares do SUS* – SIH/SUS). For this estimate, the average costs for hospitalizations that occurred in 2019 in the state of Rio de Janeiro with a primary or secondary diagnosis identified by the ICD10=“A92.0” code for Chikungunya Fever was used.

## RESULTS

The total number of CHIKF cases reported in Sinan and made available by the Rio de Janeiro municipal Tabnet (tabulation app of the Brazilian Ministry of Health) in 2019 was 38,830, and it is estimated that, among these, 20,852 progressed to the post-acute phase and 20,192 to the chronic phase. Of the reported cases, 78.98% were confirmed by clinical-epidemiological criteria, and 20.82% were confirmed by laboratory tests.

In Table 2 we describe the components of the direct costs of the acute phase and the price calculated per item for each case. The component costs of this phase are higher for the category of severe pain, as the standard treatment recommended by the Ministry of Health^2^ and SES/RJ^20^ is a combination of analgesic drugs (dipyrone and acetaminophen) and opioids (tramadol, codeine, and oxycodone). Conversely, the recommended treatment for pain of low or moderate intensity is performed only with analgesics. It is estimated that 18,250 cases were of low/moderate pain and 20,580 of severe pain. The direct costs of this phase totaled BRL 2,740,818.

**Table 2:** Components of the direct costs of the acute phase of Chikungunya for the municipality of Rio de Janeiro in the year 2019, with price per unit, amount of individual, recommended, and total doses required for treatment, and total price per case.

Item	Price	Individual dose in the treatment	Prescription in doses	Recommended total amount per case	Cost per case (BRL)
Medical appointment	BRL 23.17 per appointment	-	One every seven days	2	46.34
Minor/moderate pain treatment: dipyrone and acetaminophen interspersed					
Dipyrone	BRL 0.0774 per tabs of 500 mg	1 g (2 tabs)	6/6 h for seven days	56 tabs	4.33
Acetaminophen	BRL 0.0404 per tabs of 500 mg	500 mg (1 tabs)	6/6 h for seven days	28 tabs	1.13
Severe pain treatment: dipyrone or acetaminophen combined with an opioid					
Tramadol	BRL 0.1514 per tabs of 50 mg	50 mg (1 caps)	6/6 h for seven days	28 caps	4.24
Codeine	BRL 0.7482 per tabs of 30 mg	30 mg (1 tabs)	6/6 h for seven days	28 tabs	20.95
Oxycodone	BRL 6.3804 per tabs of 10 mg	10 mg (1 tabs)	12/12 h for seven days	14 tabs	89.33

tabs: tablet; mg: milligrams; caps: capsule.

In Table 3 we show the component costs related to the post-acute phase. The categories of pain without arthritis/nerve pain have higher costs per case than the treatment for arthritis. For pain without arthritis and nerve pain, the drugs ibuprofen, amitriptyline and gabapentin are used, while for arthritis, treatment is performed with prednisone^2,20^. The majority of cases, 15,743, are expected to progress to arthritis. Whereas 5,709 present nerve pain or pain without arthritis. Direct costs totaled BRL 1,759,900.

**Table 3:** Components of the direct costs of the post-acute phase of Chikungunya for the municipality of Rio de Janeiro in the year 2019, with price per unit, data source, amount of individual, recommended, and total doses required for treatment, and total price per case.

Item	Price	Individual dose in the treatment	Prescription	Recommended total amount per case	Cost per case (BRL)
Medical appointment	BRL 23.17 per appointment	-	One every four weeks	3	69.51
Arthritis treatment: prednisone					
Prednisone	BRL 0.1454 per tabs of 20 mg	32.5 mg (2 tabs)	32.5 mg per day for eight weeks	112 tabs	16.28
Nerve pain/pain without arthritis treatments: amitriptyline or gabapentin/ibuprofen					
Ibuprofen	BRL 0.1917 per tabs of 600 mg	600 mg (1 tabs)	600 mg 8/8h for four weeks	84 tabs	16.10
Amitriptyline	BRL 0.0379 per tabs of 25 mg	37.5 mg (2 tabs)	37.5 mg per day for two weeks	28 tabs	1.06
Gabapentin	BRL 0.3273 per tabs of 300 mg	300 mg (1 tabs)	300 mg twice a day for two weeks	28 tabs	9.16

tabs: tablet; mg: milligrams.

In Table 4 we present the outpatient costs of the components of the chronic phase treatment. Treatment is divided according to the intensity of the joint manifestation: when low, hydroxychloroquine is recommended and, when moderate/high, methotrexate combined with folic acid is recommended^2,20^. Under these conditions, the first category has the highest treatment value. It is estimated that 8,158 cases evolve with mild manifestations and 12,034 with moderate/severe manifestations, totaling BRL 4,732,986. The total direct costs, therefore, were estimated at BRL 9,233,704.

**Table 4:** Components of the direct costs of the chronic phase.

Item	Price	Individual dose in the treatment	Prescription per dose	Recommended total amount per case	Cost per case (BRL)
Medical appointment	BRL 23.17 per appointment	-	One every eight weeks	3	69.51
Treatment of mild illness: Hydroxychloroquine					
Hydroxychloroquine	BRL 1.3599 per tabs of 400 mg	325 mg (1 tabs)	One per day for six months	180 tabs	244.78
Treatment of moderate/severe illness: Methotrexate and Folic Acid					
Methotrexate	BRL 0.7629 per tabs of 2.5 mg	15 mg (6 tabs)	One per week for six months	24 tabs	109.86
Folic acid	BRL 0.0362 per tabs of 5 mg	5 mg (1 tabs)	One per week for six months	24 tabs	0.87

tabs: tablet; mg: milligrams.

To calculate indirect costs, the average daily per capita gross domestic product (GDP) of the municipality of Rio de Janeiro was used, which was estimated at BRL 134.51, considering the amount available by the IBGE, which estimated the municipal GDP per capita of BRL 49,094.40. Moreover, for the calculation of the disability component (DALY), YLL and YLD were added together. The YLD are equivalent to multiplying the number of cases that have progressed to the chronic phase, thus 20,192, by the disability weight (DW) and the duration of the disease. The DW is derived from the 2019 table of the Global Burden of Disease Study^35^, which considers the value of 0.117 for low-intensity arthritis and 0.317 for moderate intensity. The cases were divided into mild or moderate manifestation of symptoms and multiplied by the equivalent disability weight. For duration (L), 0.75 years was used in reference to the 6-month duration of the common outpatient treatment of the chronic phase considered in this study plus the 3-month duration of the post-acute phase.

In turn, the YLL were calculated by multiplying the number of deaths by the years of life lost. The years of life lost result from a decrease in life expectancy at birth in Brazil in 2019, 76.6 years, due to the individual’s age at the time of death from the disease (if the individual is over 76 years of age, their death was not considered in this calculation). In 2019, Sinan reported that 56 reported cases progressed to death by CHIKF in the municipality of Rio de Janeiro. 24 of them were individuals over 76 years of age, which does not burden the YLL. Of the remaining deaths, three were of children under 1 year of age; seven of people between 15 and 26 years of age; and the remaining were between 42 and 73 years of age. The sum of the YLDs resulted in 949.2 years. The estimate of indirect costs totaled, for the acute phase, BRL 36,561,163 and, for the post-acute and chronic phases, BRL 233,923,524.30, which together amount BRL 270,484,687.30.

For hospital costs, the number of hospitalization cases in the state of Rio de Janeiro was 256 and the average amount of HAAs was BRL 347.37. For this estimate, data from the state were used, considering that, for the municipality, the data pointed to underreporting. In addition, the municipality of Rio de Janeiro concentrates most of the state’s hospital network. Therefore, the costs result from the simple multiplication of these data, equivalent to BRL 88,926.72.

In Chart 1 we show the formulas and final results for the calculations made based on the component costs previously described. Both in direct and indirect costs, the chronic phase was the most expensive, with the indirect costs of the aforementioned phase being the highest. Hospital costs were the lowest, estimated at thousands of reais, while the others reached millions of reais. The sum of all costs was estimated at BRL 279,807,318 for the year 2019.

**Chart 1:** Calculation of the total costs of Chikungunya for the year 2019 in the municipality of Rio de Janeiro, divided into types of costs and phases of the disease, containing the expected number of cases for each category based on the notifications, the equation for the estimates, and the total in reais.

Costs	N	Equation	Total (BRL)
Direct			
Acute phase	N0=reported cases=38,830	(2 x medical appointment amount x N0) + (47% of N0 x average amount mild/moderate treatment) + (53% of N0 x average amount severe treatment) = Total	2,740,818
Post-acute phase	N1= 53.7% of N0=20,852	(3 x medical appointment amount x N1) + (75.5% of N1 x average amount treatment with arthritis) + (24.5% of N1 x amount treatment without arthritis) = Total	1,759,900
Chronic phase	N2= 52% of N0=20,192	(3 x medical appointment amount x N2) + (40.4% of N2 x mild activity treatment amount) + (59.6% of N2 x moderate/intense activity treatment amount) = Total	4,732,986
Hospital	N3=256	N3 x average HAAs	88,926.72
Indirect			
Acute phase	N0=38,830	GDPDay* x 7 x N0	36,561,163
Post-acute and chronic phases	N2=20,192	GDPYear^†^ x DALY (YLD + YLL)	233,923,524.30
DALY calculation			
DALY		949.2 + 3,815.57	4,764.77 years
YLD		[(40.4% of N2 x DW^‡^ mild^§^) + (59.6% of N2 x moderate DW^//^)] x 0.75 year	3,815.57 years
YLL		Number of deaths x life expectancy at age of death	949.2 years
Total=BRL 279,807,318)			

* GDP per capita per day;

† GDP per capita per year;

‡ disability weight;

§ 0.117;

// 0.317.

To allow the visualization of the costs for updated amounts^36^, we used an inflation correction method, considering the accumulated Extended National Consumer Price Index (*Índice de Preços ao Consumidor Amplo* – IPCA), multiplying the results by 1.2309, a value referring to the amount accumulated between 12/2019 and 12/2022 and provided by the IBGE. This correction increases the amount of costs to BRL 344,414,827.70, which would be the equivalent amount in December 2022.

Subsequently, to allow greater comparability of the results with other studies, the amount corrected by the IPCA was divided by the value of the international dollar, which considers the purchasing power parity, which, as of December 2022, was 5.164. For updated amounts, the total costs resulted in 66,695,357.81 international dollars.

## DISCUSSION

Considering the amounts of each type of cost, the intensity of the contribution of indirect costs is remarkable, accounting for 96.60% of the total costs, especially in the post-acute/chronic period, which demonstrates the burden of disability and the prevalence of symptoms in the funding of CHIKF. Authors of other studies found similar results^14-17^.

Regarding other cost studies, in the article by Teich et al.^19^, Zika and CHIKF were estimated for the state of Rio de Janeiro in 2016. The combined direct medical costs of Zika and CHIKF were calculated at BRL 9,108,866.00 and the indirect costs of CHIKF alone at BRL 8,156,401.00. In the state of Rio de Janeiro, 68,943 suspected cases of Zika and 15,383 cases of CHIKF were reported in that year. The average cost, in a simplified way, was BRL 108.00 in direct costs and BRL 530.00 in indirect costs per case, with a total average of BRL 638.00.

For the present article, the total direct costs per individual averaged BRL 390.39. In the aforementioned study, there is no distinction between the analysis of the different phases of the disease, in such a way that part of the direct costs related to the persistence of symptoms may not have been considered. If we consider only the direct costs of the acute phase, for the present study, we will have approximately BRL 71.59.

Regarding indirect costs, Teich et al.^19^ found values per individual approximately five times higher than direct values and, to calculate them, they used disability measures. In this study, the average indirect cost per individual was BRL 12,526.53, considering the three phases of the disease, and BRL 941.57, considering only the acute phase, which is equivalent to an amount 32.09 and 13.15 times higher than the direct costs, respectively. However, in our study, we included the disability component, the years of life lost related to death, which was not the case in the aforementioned article.

Cardona-Ospina et al.^16^ estimated the economic cost of CHIKF to Colombia in 2014. A total of 106,592 cases were reported during the epidemic. The total cost estimates per patient were between USD 1,438.74 and USD 3,396.57, and the total costs of the epidemic were between USD 73.6 and USD 185.5 million. In this study, considering the exchange rate at the end of 2019 of BRL 4.00 to the American dollar, the total costs of the municipal epidemic were about USD 70 million and, per case, USD 3,229.23, amounts similar to those estimated for the Colombian epidemic.

Longitudinal studies on CHIKF in the Brazilian scenario are lacking in the scientific literature. Nevertheless, the chronic phase is the most expensive and the data are insufficient to provide a detailed overview of these impacts on society and the Brazilian health system.

The present study faced methodological limitations that are characteristic of studies that estimate costs using secondary population-based data. The need to make several assumptions decreases the accuracy of the estimates. Furthermore, the absence of follow-up data for CHIKF cases in relation to the evolution of the disease and the persistence of symptoms imposed the need to use international studies to estimate the number of cases expected in each phase, which may address a different reality from the Brazilian territory.

With the introduction of CHIKF, the municipality of Rio de Janeiro found the possibility of exacerbating its historic challenges in relation to the circulation of arboviruses. The conditions of the territory are fruitful for the disease to continue to have an impact on the population. Therefore, strategies to combat the vector become even more relevant, considering their effectiveness in reducing the circulation of all these arboviruses. The dynamics of these vectors in the territory have a significant impact on public spending and are reflected in the overburden of the healthcare system. That is why it is important to direct actions to prevent and combat diseases and their vectors.

The CHIKF epidemic in 2019 in the municipality of Rio de Janeiro generated significant costs, mainly due to the persistence of joint symptoms in the chronic phase, as disability is the biggest cost generator of the disease. This characteristic sets CHIKF apart from other arboviruses.
